# Unmet needs in patients with acute myeloid leukemia ineligible for intensive approaches: perspectives from a European expert panel

**DOI:** 10.3389/fonc.2025.1642472

**Published:** 2025-10-31

**Authors:** Felicetto Ferrara, Klaus Geissler, Priyanka Mehta, Pau Montesinos, Adriano Venditti

**Affiliations:** ^1^ Hospital Antonio Cardarelli, Naples, Italy; ^2^ Sigmund Freud Private University, Vienna, Austria; ^3^ University Hospitals of Bristol and Weston NHS Trust, Bristol, United Kingdom; ^4^ La Fe University and Polytechnic Hospital, Valencia, Spain; ^5^ Hematology, Department of Biomedicine and Prevention, University of Rome Tor Vergata, Rome, Italy

**Keywords:** acute myeloid leukemia, age, outcome, fitness, quality of life

## Abstract

Acute myeloid leukemia (AML) is primarily a disease of the elderly, with increasing age associated with worse outcomes. Treatment options include intensive chemotherapy, hypomethylating agents with/without venetoclax, and best supportive care. Although the treatment landscape for AML has progressed in recent years, survival in older, frail patients ineligible for intensive chemotherapy remains poor. To address this, a panel of European experts convened to discuss the key factors influencing AML prognosis in older patients and/or those deemed unfit for intensive therapy. The panel shared perspectives on AML outcomes, patient fitness, treatment choices, and unmet needs. Experts agreed that although age is an important factor in guiding therapeutic decision making, other factors should also be considered such as comorbidities and the impact of disease biology (e.g., cytogenetic/molecular aberrations). Experts also agreed that more robust assessments of patient fitness are required, such as the utilization of geriatric assessment tools. As choice of therapy and its associated toxicities can impact patient’s quality of life (QoL), the logistical, psychosocial, and financial challenges experienced by the patient and their support network needs to be considered when determining treatment. Finally, experts agreed that outcomes in older, unfit patients with AML remain suboptimal in part due to the impact of treatment-related toxicities and QoL burden. There is therefore an urgent need for alternative treatments which minimize toxicity and reduce QoL burden.

## 1 Introduction

Acute myeloid leukemia (AML) is a rare hematopoietic disorder, accounting for around 1% of all cancers (1). It is characterized by infiltration of the bone marrow, blood, and other tissues by proliferative, clonal, abnormally differentiated/undifferentiated hematopoietic cells (2). Symptoms include fatigue, recurrent infections, and bleeding which progress and worsen over time (3). Whilst AML can occur in any age group, it is primarily a disease of the elderly and increasing age is associated with worse outcomes (4–6). Given its acute nature, AML can progress quickly and aggressively, usually requiring immediate treatment (7).

Patients with AML have access to multiple treatment options spanning a broad spectrum, from intensive to less intensive therapy. Treatment regimens such as intensive chemotherapy aim to control and eliminate the disease by inducing a complete response with initial therapy (e.g., cytarabine + anthracycline), followed by consolidation with intermediate- or high-dose cytarabine (8). Maintenance treatment with a hypomethylating agent (HMA; e.g., azacitidine) is used to prolong remission and maximize response duration in patients with intermediate or adverse risk disease who are not candidates to receive allogeneic hematopoietic cell transplantation (alloHCT). For patients with particular molecular aberrations, targeted therapies may be an additional treatment option. For patients considered unfit for intensive chemotherapy, treatment options are typically limited to HMAs with venetoclax or HMAs alone, or best supportive care (BSC), depending on their level of frailty as judged by the physician, as well as the patient’s preference. Less intensive treatment options do not have a curative intent but rather aim to prolong survival and preserve quality of life (QoL).

Despite the variety of treatment options available, over 50% of newly diagnosed patients with AML are not considered for intensive induction treatment due to several factors including age, comorbidities, and Eastern Cooperative Oncology Group (ECOG) performance status (PS) (7). Consequently, outcomes vary and 5-year overall survival (OS) is lower in patients aged >60 years (~17%) compared with those aged <60 years (55%). As therapies for patients considered unfit for intensive chemotherapy or alloHCT are more limited and outcomes are suboptimal, there is an urgent need for better treatment options for an increasingly aging population. To address this, a panel of five European experts from Austria, Italy, Spain, and the UK, was established to discuss the key factors influencing AML including outcomes, treatment options for older patients and/or those deemed unfit for intensive therapy, and unmet needs. Members of the expert panel participated in a virtual consensus meeting on 3^rd^ October 2024 to discuss these topics. Herein, we share perspectives from the European expert panel, including defining patient fitness for treatment, the importance of biological factors on treatment choice, and patient QoL measures.

## 2 AML outcomes in older, unfit patients

### 2.1 Expert panel perspectives

- Outcomes in older patients with AML are poor, and prognosis worsens with age
- Despite treatment advances in AML, therapeutic options for older, unfit patients are less effective compared to younger patients, and outcomes remain unsatisfactory

AML is the most common acute leukemia in adults with a median age of diagnosis of 68 years (9). AML incidence rates increase with age, with 1.4 cases per 100,000 patients in younger adults (<50 years; 2015) compared with 20.3 and 27.3 cases per 100,000 patients in the 65+ and 75+ age categories, respectively (5). Increasing age is associated with worse outcomes for AML (4, 5). Older patients (>60 years) have poor long-term survival rates, with a median OS of 10 months, compared with around 24 months for younger patients (<60 years) (6). Complete remission (CR) rates following intensive therapy are also lower in older patients (40–55%) compared to younger patients (60–80%) (6, 10).

Treatment advances for AML have been made over recent years, particularly with regards to alloHCT techniques and advancements in supportive care for intensive chemotherapy (11, 12). This has resulted in improved outcomes in younger, fit patients with 5-year survival rates in patients aged <60 years with *de novo* AML increasing from 13% in the 1970s, to 55% in the 2010s (13). However, similar improvements in outcome have not been observed in the older population (14) where these options are rarely recommended to patients >70 years. For example, in the UK National Cancer Research Institute AML18 trial, only 4% of patients who received an alloHCT were aged >70 years (15).

The treatment landscape for older, unfit patients with AML substantially changed following the approval of venetoclax in combination with a HMA for the treatment of newly-diagnosed AML in patients ineligible for intensive chemotherapy (16). In a Phase 1 study, 67% of patients achieved CR (+ CR with incomplete count recovery) with venetoclax plus decitabine or azacitidine (17) and this combination was well tolerated in high-risk groups, including patients aged >75 years, and those with poor genetics or secondary AML (17). The Phase 3 VIALE-A trial later demonstrated that patients treated with venetoclax in combination with azacitidine had significant improvements in median OS (14.7 months) compared to those who received azacitidine alone (9.6 months) (18). The development of targeted agents, e.g., ivosidenib and enasidenib for patients with *IDH1/2* mutated AML, and gilteritinib for patients with *FLT3*-mutated AML, has also offered options to older, unfit patients who would have otherwise been offered BSC (12).

Despite these advances, the expert panel agreed that therapeutic options for older, unfit patients are less effective compared to younger patients, and outcomes remain unsatisfactory. Real-world studies have been unable to demonstrate similar OS rates as seen in clinical studies with substantially lower OS rates (~10 months) observed in meta-analyses comparing real-world data to clinical studies such as VIALE-A (19, 20). Therefore, further studies evaluating this variance are required to determine whether this is due to differences in patient characteristics or non-optimal adherence to therapy. New therapies which aim to reduce the toxicity and QoL burden associated with current treatment options whilst improving clinical outcomes such as OS are urgently needed for older, unfit patients with AML. Furthermore, the inclusion of older, unfit patients in clinical trials is critical to ensure the development and assessment of therapies in this difficult to treat population.

## 3 How patient fitness can be used to determine treatment choice

### 3.1 Expert panel perspectives

- Choice of intensive versus non-intensive therapy depends on fitness considerations, mostly in older patients
- Fitness considerations, including age, performance score, comorbidities and physical and cognitive function, should be considered alongside patient preference when determining the most appropriate treatment

Assessment of ‘fitness’ is an important consideration when determining the appropriate treatment strategy for patients with AML. Although age is an important factor, it is important to assess other patient factors such as comorbidities, ECOG PS, physical, cognitive and psychosocial function, as well as characteristics of their disease, e.g., cytogenetic/molecular aberrations and white blood cell count (21, 22). A recent panel consensus on behalf of the European LeukemiaNet (ELN) recommended that a comprehensive, patient-centered evaluation of fitness factors should be conducted before initiating any therapeutic regimen (23). In addition, as a patient’s fitness status can change during treatment, it should be reassessed before each treatment phase. However, there are currently several risk scoring systems for predicting treatment tolerance and a lack of generally accepted or validated criteria to consider a patient ineligible for intensive chemotherapy (8). Existing tools include the Ferrara criteria, hematopoietic stem cell transplantation-comorbidity index (HCT-CI), and Charlson comorbidity index (CCI) (21, 24, 25). In a large real-world study of older patients with AML, higher CCI independently predicted poorer survival (26). HCT-CI was developed to improve the sensitivity of the CCI in the alloHCT setting and a retrospective study of older patients with AML receiving intensive chemotherapy showed a correlation between higher HCT-CI scores and earlier death rates (27, 28).

Although these tools may stratify some patients as unfit for intensive treatment, there are cases where seemingly fit patients without relevant comorbidities can have considerable functional or cognitive impairment (12). Geriatric assessments are crucial for detecting these impairments and can discriminate fit from unfit patients as demonstrated in a study by Klepin et al. (29). This study identified significant impairment and heterogeneity in physical, cognitive, and psychological health in older patients with AML who were considered fit for intensive chemotherapy by standard oncology assessments, and highlighted that these factors were more important than chronological age in predicting survival. More recently, Bhatt et al. used geriatric assessments to capture variations in multidimensional health (22) and concluded that understanding the risk of mortality and treatment tolerance can better inform patients of anticipated outcomes after treatment and may facilitate advance care planning. They suggested that geriatric assessments should be completed before or within a few days of initiation of chemotherapy and include, at a minimum, measures of comorbidity burden, cognition, physical function, and emotional health (22).

**Figure 1:**
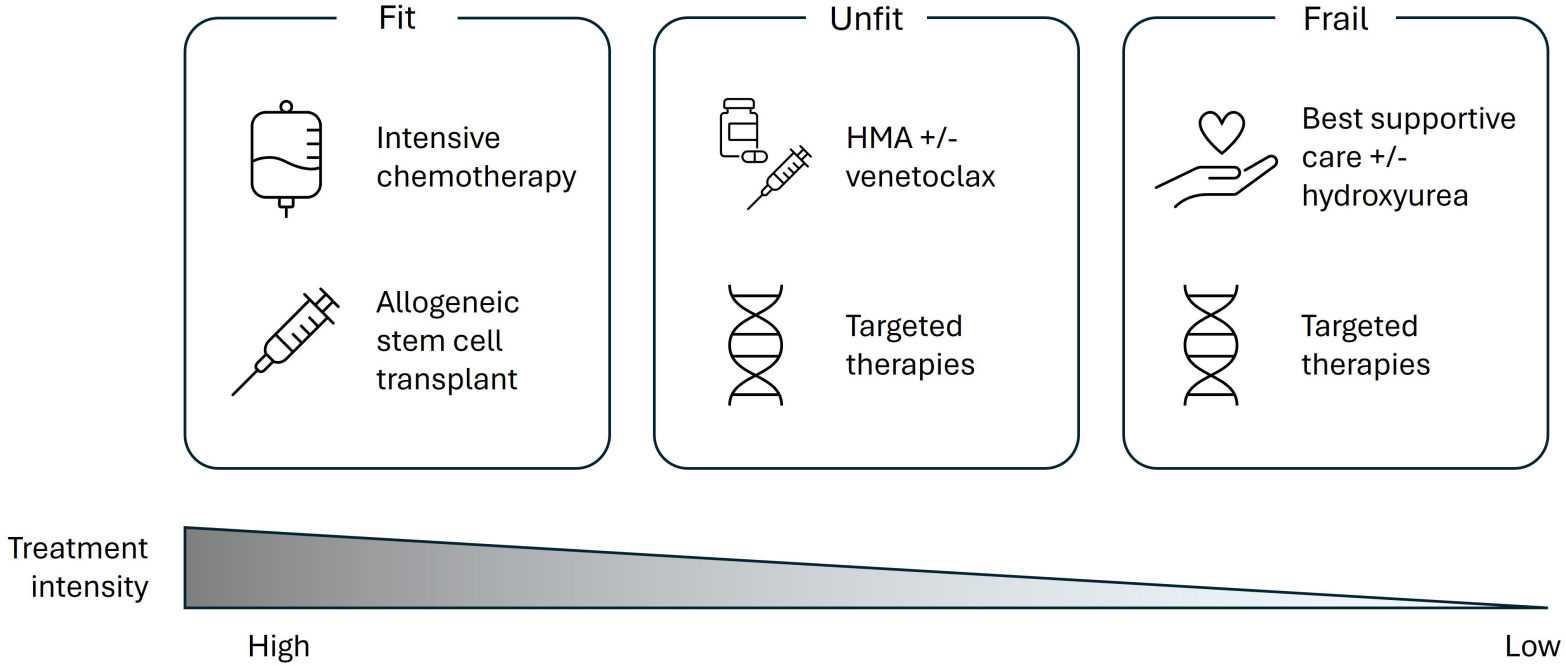
Current perspectives of treatment options across the patient fitness spectrum according to a European expert panel. HMA, hypomethylating agent.

Based on this evidence and further discussion, the expert panel agreed that clinicians should utilize geriatric assessment tools to examine patient fitness as previous studies have highlighted the importance of these assessments in determining treatment tolerance. For older patients who undergo a geriatric assessment, the expert panel suggested that treatment choice for these patients should be determined according to their fitness on a spectrum: fit, unfit, or frail (**Figure 1**), whilst also considering patient preference and QoL when determining the optimal treatment approach.

Prognostic scores incorporating disease biology, patient fitness, and comorbidities, that can be used to calculate long-term survival would also be of benefit and efforts should be made to consider developing this approach in the future. There are ongoing initiatives through ELN to provide clearer guidance on criteria for fitness/unfitness. However, recent ELN risk stratification systems are based on response to intensive chemotherapy and studies have highlighted that this model has limited applicability in older patients given lower-intensity treatment (8, 30, 31).

## 4 The importance of biological factors on treatment choice

### 4.1 Expert panel perspectives

• Biology of the disease is an important factor that should be considered when determining the most appropriate treatment

Mutational profiling has significantly advanced the understanding of the molecular pathogenesis of AML. Several molecular aberrations, e.g., driver mutations in *FLT3*, *IDH1*, and *IDH2*, have been identified and adverse cytogenetic abnormalities have been shown to increase with age. For patients with favorable-risk disease based on genetic risk classification, median OS of >24 months has been reported; however, this decreases to 5–8 months for patients with adverse-risk disease (32). Whilst ELN genetic risk classifications have been widely used in clinical practice to stratify prognostic outcomes for patients with AML (4, 8, 31), they are suboptimal for older patients as they were based exclusively on data from patients receiving intensive chemotherapy (4, 8). Consequently, new recommendations on genetic risk classification for patients with AML receiving less-intensive therapies have been developed (31). Within these recommendations, patients with mutations in *NPM1*, *IDH1*, *IDH2*, and *DDX41* are categorized as favorable risk, with median OS ranging from 23–39 months. Patients with *FLT3*-*ITD^pos^* and/or *NRAS^mut^* and/or *KRAS^mut^* tumors have an intermediate risk (OS ~12 months), whereas those with *TP53-*mutated tumors are associated with adverse clinical risk (OS 5–8 months) (31). Considering these developments in understanding AML biology and the associated prognostic risk, and availability of drugs which target specific driver mutations (e.g., *FLT3*, *IDH1*, and *IDH2 (* 33), the experts agreed that the impact of disease biology should be considered alongside other factors like age, geriatric assessments, comorbidities etc. when determining an appropriate therapeutic approach (34). For example, for fit patients with *FLT3*-mutated AML, their preferred option may be intensive chemotherapy combined with a FLT3 inhibitor, e.g., midostaurin or quizartinib, which has shown significant improvements in outcomes in both younger and older patients (8, 35). For older, unfit patients, biological information can also be important in determining between venetoclax + HMA or HMA alone, particularly for patients with *TP53* mutations. Patients with *TP53* mutations/complex karyotype or a monocytic phenotype, have poor outcomes with HMA alone or in combination with venetoclax (31, 36, 37). Therefore, the experts suggested that the preferred treatment may be HMA monotherapy to decrease the level of toxicity. Patients with *IDH1* mutations are eligible for HMA + ivosidenib or HMA + venetoclax (12, 38).

Despite the importance of cytogenetic factors, the expert panel agreed that it is currently difficult to make treatment decisions solely based on biological factors, with a lack of standardized guidance on how to treat patients with different mutations, with the exception of few mutations with targeted treatments. Another limitation is the timescale of molecular testing via next-generation sequencing (NGS), which can take between 1–4 weeks. For some patients with indolent disease (i.e., where the disease progresses slowly without significant symptoms), delaying treatment until NGS results are available, would be acceptable. However, for patients with progressive disease, a shorter turnaround time of mutation test results would be vital for the initiation of appropriate treatment. Experts agreed that there is an urgent need for a well-organized centralized system to reduce result turnaround time, which currently varies from country to country.

## 5 Toxicity and quality of life burden with existing treatments

### 5.1 Expert panel perspectives

• In older, unfit patients, non-intensive therapies can still be associated with significant toxicity and a substantial quality of life burden

Toxicities associated with both intensive and less intensive treatments can impact patient QoL and treatment choice. Whilst toxicities with intensive treatments have been well established, significant AEs have been reported with less intensive, combinations treatments. For instance, in the VIALE-A trial, there was a higher rate of serious AEs reported (e.g., febrile neutropenia and pneumonia) with the combination of venetoclax plus azacitidine compared with azacitidine plus placebo (18). In addition, there was a higher incidence of dose interruption in the combination group and the majority of these patients had modifications to the duration of venetoclax, with some also receiving granulocyte colony-stimulating factor during remission. Supportive care measures, including the addition of antibiotic, antiviral, and antifungal therapy, were also recommended for patients receiving the combination therapy. The expert panel highlighted the management of toxicities with venetoclax as an area of improvement and suggested that appropriate use of venetoclax, e.g., with the use of a de-escalation or reduction scale, can allow clinicians to manage toxicities more appropriately and potentially limit these to the initial course of venetoclax + HMA.

The expert panel also agreed that it is important to consider the logistical, emotional and financial challenges experienced by not only the patient, but also their support network and caregivers as these can impact QoL. The Acute Leukemia Advocates Network (ALAN) survey identified body pain and skin issues as key detrimental side effects that affect QoL (34). Hospitalization and access to treatment takes up a considerable amount of time for patients with AML, and more than 80% of patients surveyed reported having visited or stayed in hospital for between 1 and 7 days in the previous month (34). The majority of patients stated a travel time of between 30 minutes and 2 hours from their home to the hospital, with almost 10% of patients reporting that the journey takes over 2 hours. This is of particular importance for patients receiving treatment with HMAs, which are typically administered parenterally for 5–7 days per treatment cycle, with multiple cycles normally required for a maximal clinical response. This can have a significant impact on a patient and their caregiver due to the time spent travelling to, preparing for, and receiving intravenous (IV) treatment (34, 39). Spouses/partners are the main caregivers for more than 70% of older patients, with the level of help required dependent upon the disease stage (34). Many patients rely on family and friends for their everyday needs, with most patients reporting that their disease had an emotional impact on their caregivers, for example the burden of frequent hospital visits (34, 40). Recently, an expert panel recommended that patient social support should be intensified when considering any therapeutic interventions, particularly in the absence of caregivers (23).

The inconvenience and burden of travelling to appointments for IV infusions also supports the need for oral treatments that can be administered in the patient’s home (34). A recent study explored the preference of HMA mode of administration in patients with AML with most patients preferring oral administration over parenteral routes due to convenience. However, treatment efficacy and associated side effects were key factors to consider when deciding on a treatment approach. Oral HMA treatment with equivalent efficacy and tolerability might decrease the burden of parenteral treatment and improve patient QoL (40).

In addition to toxicity burden, the ALAN survey also highlighted the emotional and financial impact treatment has on patients. Many patients reported that they experienced financial difficulties as a result of their diagnosis, more often during treatment or relapse, compared to remission (34). AML treatments that induce and prolong remission may therefore reduce healthcare resource utilization and the economic burden of disease (41). Based on this information and discussions with the expert panel, there is a need for more effective treatments with lower toxicities that improve the QoL burden for patients.

## 6 Unmet needs and future directions

### 6.1 Expert panel perspectives

• Alternative, non-intensive treatment options which are less toxic and have less impact on quality of life are required for older, unfit patients ineligible for intensive chemotherapy

Evidence from this expert panel demonstrates that outcomes in older, unfit patients with AML remain suboptimal and there is an urgent need for alternative treatments due to the impact of treatment-related toxicities and QoL burden. This is especially true for older, unfit patients, particularly those deemed ineligible for HMA + venetoclax. Several therapies are currently under investigation for AML (**Table 1**); although, detailed discussion on these was not part of the expert panel meeting. However, the expert panel did discuss combination treatments with decitabine. Whilst they were supportive of the combination of decitabine with cedazuridine, experts felt that there was insufficient evidence to support the combination of oral decitabine with venetoclax at the time of the consensus meeting. However, recently reported Phase 1/2 clinical trial results in patients ineligible for intensive chemotherapy show promising efficacy and safety results (42–44). Experts also identified other patients who may benefit from new therapies; including those with myelodysplastic syndrome (MDS) or myeloproliferative neoplasms (MPN) who transformed to AML and may not tolerate significant myelotoxicity.

**Table 1:** Investigational therapies for AML.

Type	Class/pathway	Agents
Targeted therapies	FLT3 inhibitor	Crenolanib, Ponatinib, Luxeptinib, HM43239, FF-10101
	IDH inhibitor	LY3410738
	BCL2 inhibitor	S65487, S55746
	MCL1inhibitor	AMG 176, AZD5991, S64315
	Mutant P53	Eprenetapopt
Epigenetic pathways	Menin-MLL inhibitor	Ziftomenib
	Menin inhibitor	Revumenib, bleximenib
	DOT1L inhibitor	Pinometostat
	KDM1A inhibitor	Iadademstat
	HDAC1/3 inhibitor	Entinostat
Oncogenic pathways	NEDD8 activator	Pevonedistat
	Syk inhibitor	Entospletinib
	PARP inhibitor	Olaparib
	MDM2/HDM2 inhibitor	Milademetan, Siremadlin
	E-selectin antagonist	Uproleselan
	Retinoic acid receptor alpha agonist	Tamibarotene
	Dihydroorotate dehydrogenase inhibitor	JNJ-74856665
	PRMT5 inhibitor	PRT543
	Splicing modulator	H3B-8800
	IRAK4 inhibitor	Emavusertib
Targeted immune inhibitors	Anti-CD47	Lemzoparlimab
	Anti-CTLA4	Ipilimumab
	Anti-PD-1	Nivolumab, Pembrolizumab, Spartalizumab
	Anti-LAG3	Relatlimab
	Anti-TIM3	Sabatolimab
Decoy receptor	Anti-CD47	Evorpacept
Antibody-drug conjugate	Anti-CD123	IMGN632
	Anti-CD70	Cusatuzumab
	Anti-CD33	Vadastuximab
Bispecific T-cell engager/dual-affinity retargeting protein	CD33 x CD3	AMV564
	FLT3 x CD3	AMG 427
	CD123 x CD3	Flotetuzumab, Vibecotamab, APVO436

Table adapted from Bhansali et al., 2023 (45).

## 7 Conclusions

AML is a disease of the elderly with age and genetics being important risk factors for prognosis. Treatment options for older patients who are unfit for intensive chemotherapy include HMA with/without venetoclax and targeted agents. However, outcomes remain suboptimal for these patients. The expert panel agreed that fitness considerations for treatment are important, and geriatric assessments are vital measures that could improve prognosis. Genetics is an important factor which must be considered, particularly for *FLT3*, *IDH1/2*, and *TP53* mutations; however, these cannot be considered in isolation. Improved treatment options which minimize toxicity and reduce QoL burden are urgently needed for older, unfit patients with AML.

## Data Availability

The original contributions presented in the study are included in the article/supplementary material. Further inquiries can be directed to the corresponding author.
